# Challenges documenting racial disparities in Merkel cell carcinoma

**DOI:** 10.1080/15384047.2022.2145842

**Published:** 2022-11-20

**Authors:** Mackenzie R. Martin, Noreen Mohsin, Serena Vilasi, Danielle Reed, Isaac Brownell

**Affiliations:** Dermatology Branch, National Institute of Arthritis and Musculoskeletal and Skin Diseases, Bethesda, MD, USA

**Keywords:** Merkel cell carcinoma, health disparities, racial disparities, immunotherapy, survival, tumor registries, skin cancer, rare diseases

## Abstract

Merkel cell carcinoma (MCC) is a rare, aggressive neuroendocrine skin cancer that predominantly impacts White patients. Overall incidence and the proportion of minority patients with MCC are both rising. In the more common skin cancer, melanoma, racial disparities are well-documented in stage at presentation and patient survival. Whether racial and ethnic disparities exist in MCC remains unclear. The study of MCC disparities is hampered by limitations in data registries, including SEER and NCDB, and an evolving natural history due to the advent of immunotherapy. Published MCC immunotherapy clinical trials consistently reported the racial diversity among enrolled subjects but failed to include patients’ ethnicities. Efforts to improve data capture in cancer registries and create multi-institutional clinical databases will allow for more effective study of racial and ethnic disparities in rare cancers like MCC. Such studies are needed to advance policies promoting equity in care.

Merkel cell carcinoma (MCC) is a rare, aggressive neuroendocrine skin cancer with an overall incidence of 0.7 cases per 100,000 person-years in the U.S.^1^ Locoregional MCC is treated with surgery and radiotherapy whereas advanced MCC is treated with immunotherapy.^1^ MCC predominantly affects non-Hispanic White patients with an incidence of 0.8. The estimated incidence for Black and Hispanic patients is 0.1 and 0.3, respectively.^1^ Melanoma, another skin cancer predominantly affecting White patients, is about thirty times more common than MCC.^1,2^ Black melanoma patients have worse survival and present more often with advanced disease than White patients, suggesting possible health inequities.^3^ Thus, studies investigating potential disparities in MCC are warranted.

Consideration of racial and ethnic disparities in skin cancers is a complex issue. Race and ethnicity are social constructs that correlate with socioeconomic status and beget institutional biases that contribute to healthcare inequities. Identification with a racial or ethnic group can be influenced by skin color and geographical ancestry which, in turn, can correspond to genetic haplotypes and biological responses to ultraviolet light that influence skin cancer risk. Understanding these relationships will improve skin cancer management and help build equity in healthcare structures. Racial and ethnic disparities in MCC are typically studied through analyses of the two large U.S. cancer registries: Surveillance, Epidemiology, and End Results Program (SEER) and the National Cancer Database (NCDB).

Whether race and ethnicity influence MCC stage and outcome is unclear. Analysis of SEER 17 (1988–2012) and NCDB (2004–2013) determined that Black MCC patients present more often with distant metastases than White MCC patients.^4,5^ In contrast, analysis of SEER 9 (1973–2011) did not find an association between advanced stage and Black race.^6^ Given Black race is associated with lower income in the U.S., it is important to consider that patients with lower income also present more often with advanced MCC and have worse survival.^6,7^ Although the use of radiotherapy and surgery amongst MCC patients does not differ by race or income, wait times to treatment does differ.^7,8^ This suggests treatment latency may contribute to MCC disparities.

Kaplan-Meier analysis of SEER 9 (1973–2011) and SEER (1986–2013) determined that Black MCC patients’ survival is significantly lower than their White counterparts.^6,7^ Analysis of SEER 9 (1973–2011) and SEER (1986–2013) that controlled for stage, primary site, and socioeconomic status found that Black MCC patients face lower overall survival rates than White MCC patients.^6,7^ However, multivariate analysis of data from SEER 17 (1988–2012) and NCDB (2004–2016) failed to establish an association between race and survival.^4,9^ Studies to resolve these inconsistencies and better understand MCC disparities are needed.

Due to the rarity of MCC, cases spanning many years are needed to obtain an adequate number of observations. Over time, diagnosis standards and disease management can evolve. Specifically, immunotherapy use is a recent therapeutic shift that is changing the natural history of MCC.^1^ Such changes result in heterogenous datasets and add potential confounders to analyses. In addition to small sample sizes and changing natural history, inconsistencies in how race and ethnicity impact MCC outcomes may be impacted by registry limitations.

Selection biases and missing data may explain some contradictory results on MCC disparities. SEER features several versions that represent between 9% to 48% of the U.S. population. Potential biases in SEER include an overrepresentation of foreign-born patients, urban inhabitants, and the proportion of Hispanic and Asian/Pacific Islander to White and Black patients.^10^ SEER data collection also favors high-volume medical centers, resulting in a greater proportion of patients with late-stage cancers.^10^ Moreover, statistics on comorbidities, immunotherapy use, and disease recurrence are not recorded; and only summary staging information is available for ~80% of MCC cases.^4,6,7^ These limitations must be considered when extrapolating data from SEER.

The NCDB has its own potential limitations. Collecting data from over 1,500 Commission on Cancer (CoC) facilities, NCDB represents more than 72% of diagnosed U.S. cancers. CoC hospitals are larger, more urban, and offer more services, subjecting the NCDB to selection biases.^11^ Longitudinal NCDB data are heterogenous as participating hospitals change annually. The NCDB also lacks disease-specific survival and recurrence data, restricting outcomes analysis.^12^ Additionally, 22% of MCC cases with follow-up data failed to include stage information.^12^ Understanding registry shortcomings allows for a more complete contextualization of analysis on disparities.

Despite limitations, both data registries adequately capture race and ethnicity data. For MCC cases in SEER from 1973 to 2013, 1% to 3% were missing race and 3% were missing ethnicity.^4,6,7^ Of NCDB MCC cases diagnosed from 2004 to 2013, 1% and 6.1% were missing race and ethnicity, respectively.^5^

As new cancer therapies emerge, diversity and inclusion among clinical trial subjects is a major concern. Searching ClinicalTrials.gov (March 29^th^, 2022), we identified four MCC immunotherapy clinical trials having enrolled more than five participants with published results (Supplemental Methods; Supplemental Figure 1). All four studies reported race information and the aggregate proportions of Black, AAPI, and White patients were congruous with the reported incidence of MCC cases across racial groups in the U.S. (Table 1). However, race was unknown for 14.6% of subjects, and no study tracked ethnicity. Despite these limitations, it appears that MCC immunotherapy trials have been racially representative in their patient enrollment – an encouraging achievement for a rare cancer.

**Table 1.** Racial and ethnic representation in Merkel cell carcinoma clinical trials and the U.S.

*Merkel Cell Carcinoma Immunotherapy Clinical Trials*							
CT. Gov Identifier	Intervention	Total (N)	Unknown (N)	White (N)	Black (N)	AAPI (N)	Other (N)
NCT02584829	XRT, IFN-β and Avelumab w/ or w/o Cellular Adoptive Immunotherapy	8	0	8	0	0	0
NCT02267603	Pembrolizumab	50	4	45	0	1	0
NCT02488759*^13^	Nivolumab	39	0	36	2	0	1
NCT02155647*	Avelumab	204	40	156	2	6	0
MCC Immunotherapy Clinical Trials Total (N = 257)				245 (95.3%)	4(1.6%)	7(2.7%)	1(0.4%)
*US Population Merkel Cell Carcinoma cases*							
Registry (Year)				White (N)	Black (N)	AAPI (N)	Other (N)
SEER-9 (1973–2011) (N = 2675)^6^				2,544 (95.1%)	32 (1.2%)	92 (3.4%)	7 (0.3%)
NCDB (2004–2013) (N = 8673)^5^				8,461 (97.6%)	113 (1.3%)	59 (0.7%)	40 (0.5%)

*study enrolled in international sites

Abbreviations: CT. Gov Identifier, ClinicalTrials.Gov Identifier. AAPI, Asian American or Pacific Islander. XRT, localized radiation therapy. IFN-β, recombinant interferon beta.

Improving equity of MCC treatment will require rigorous study that acknowledges shortcomings in the data. To reduce bias and increase representation, registries should be expanded to include diverse data sources. Improved data capture quality control may address missing information. Expanding details on treatment histories will be needed to better characterize differences in outcomes between racial and ethnic groups. To expand research beyond cancer registries, institutions with high volumes of MCC patients could collaborate to develop a detailed clinical database. Finally, MCC clinical trials must strive to continue to enroll diverse participants and include patient race and ethnicity as study variables. These efforts may advance equitable improvements in MCC care.

## Supplementary Material

Supplemental Material

## Data Availability

Data derived from https://clinicaltrials.gov in March of 2022.
