# Supplementary material for: Challenges documenting racial disparities in Merkel cell carcinoma
Source: Cancer Biol Ther. 2022 Nov 20;23(1):1–3. doi: 10.1080/15384047.2022.2145842 (PMC9683062; doi:10.1080/15384047.2022.2145842)
Supplement: Supplemental Material [file KCBT_A_2145842_SM3117.docx]

**Supplemental Material**

**Challenges documenting racial disparities in Merkel cell carcinoma**

Mackenzie R. Martin*^1^,Noreen Mohsin *^1^, Serena Vilasi^1^, Danielle Reed^1^, and Isaac Brownell, MD, PhD^1^.

*Contributed Equally

^1^Dermatology Branch, National Institute of Arthritis and Musculoskeletal and Skin Diseases, NIH, Bethesda, MD.

**Supplemental Methods**

**Supplementary Figure 1 – Flow diagram of studies using immunotherapy for Merkel cell carcinoma included in analysis**

**Supplemental Methods**

We searched ClinicalTrials.gov and PubMed for clinical trials investigating the use of immunotherapy in patients with MCC. Search terms included "Merkel cell carcinoma" and "immunotherapy", "avelumab", "pembrolizumab", or "nivolumab." 35 trials were identified using the search terms “Merkel cell carcinoma” and “immunotherapy”. 16 trials were identified using the search terms “Merkel cell carcinoma” and “avelumab”. 23 trials were identified using the search terms “Merkel cell carcinoma” and “pembrolizumab”. 14 trials were identified using the search terms “Merkel cell carcinoma” and “nivolumab”.  We included trials that were recruiting; enrolling by invitation; active, but not recruiting; suspended; terminated; completed; withdrawn; or had an unknown status. Trials were excluded if they were unrelated to Merkel cell carcinoma or trials if less than 5 participants were enrolled.


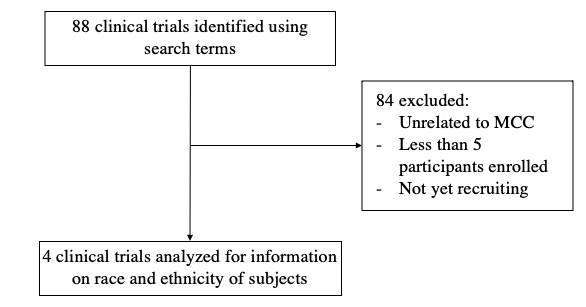


**Supplementary Figure 1: Flow diagram of studies using immunotherapy for Merkel cell carcinoma included in analysis.** MCC, Merkel cell carcinoma.
